# Hsp90β inhibition modulates nitric oxide production and nitric oxide-induced apoptosis in human chondrocytes

**DOI:** 10.1186/1471-2474-12-237

**Published:** 2011-10-17

**Authors:** Valentina Calamia, Maria C de Andrés, Natividad Oreiro, Cristina Ruiz-Romero, Francisco J Blanco

**Affiliations:** 1 Rheumatology Division, ProteoRed/ISCIII Proteomic Group, INIBIC - Hospital Universitario A Coruña, As Xubias S/N, 15006 - A Coruña, Spain; 2 Rheumatology Division, INIBIC - Hospital Universitario A Coruña, As Xubias S/N, 15006 - A Coruña, Spain; 3 CIBER-BBN, Instituto de Salud Carlos III, A Coruña, Spain

## Abstract

**Background:**

Hsp90β is a member of the Hsp90 family of protein chaperones. This family plays essential roles in the folding, maturation and activity of many proteins that are involved in signal transduction and transcriptional regulation. The role of this protein in chondrocytes is not well understood, although its increase in osteoarthritic cells has been reported. The present study aimed to explore the role of Hsp90β in key aspects of OA pathogenesis.

**Methods:**

Human OA chondrocytes were isolated from cartilage obtained from patients undergoing joint replacement surgery, and primary cultured. Cells were stimulated with proinflammatory cytokines (IL-1β or TNF-α) and nitric oxide donors (NOC-12 or SNP). For Hsp90β inhibition, two different chemical inhibitors (Geldanamycin and Novobiocin) were employed, or siRNA transfection procedures were carried out. Gene expression was determined by real-time PCR, apoptosis was quantified by flow cytometry and ELISA, and nitric oxide (NO) production was evaluated by the Griess method. Indirect immunofluorescence assays were performed to evaluate the presence of Hsp90β in stimulated cells.

**Results:**

Hsp90β was found to be increased by proinflammatory cytokines. Inhibition of Hsp90β by the chemicals Geldanamycin (GA) and Novobiocin (NB) caused a dose-dependent decrease of the NO production induced by IL-1β in chondrocytes, up to basal levels. Immunofluorescence analyses demonstrate that the NO donors NOC-12 and SNP also increased Hsp90β. Chemical inhibition or specific gene silencing of this chaperone reduced the DNA condensation and fragmentation, typical of death by apoptosis, that is induced by NO donors in chondrocytes.

**Conclusions:**

The present results show how Hsp90β modulates NO production and NO-mediated cellular death in human OA chondrocytes.

## Background

Osteoarthritis (OA) is a slowly progressive degenerative disease characterized by the degradation of the extracellular matrix (ECM) and cell death, resulting in a gradual loss of articular cartilage integrity, intra-articular inflammation and changes in peri-articular and subchondral bone [1]. The chondrocyte is the only cell type present in mature cartilage and is responsible for repairing the cartilage tissue damaged by OA.

Chondrocytes are key players in the control of cartilage matrix turnover through the production and secretion of collagens, proteoglycans, and enzymes affecting cartilage metabolism [2]. Chondrocyte metabolism is influenced by several cytokines and growth factors, which drive two qualitatively distinct functional programs in these cells: the catabolic program is induced by proinflammatory stimuli and characterized by the secretion of proteases, suppression of matrix synthesis, and induction of chondrocyte apoptosis. The anabolic program is associated with the secretion of cytokines antagonistic to the catabolic program, synthesis of protease inhibitors, production of ECM, and cell replication [3]. The balance between these processes is essential for a proper tissue turnover, and efforts should focus on this issue in order to gain a better understanding on OA pathogenesis and be able to develop new therapy strategies.

Following this reasoning, we performed a differential proteomic analysis in order to search for OA-related changes in human articular chondrocyte intracellular proteins, which aimed to unravel those molecular mechanisms that participate in OA pathogenesis [4]. Among the proteins that were significantly altered in OA chondrocytes, we identified the β subunit of the chaperone Hsp90 as increased in diseased cells. This increase was verified by immunodetection methods both in OA chondrocytes and cartilage, and we found that the proinflammatory cytokine IL-1β acts as a positive modulator of Hsp90β abundance [4].

Hsp90β is a member of the Hsp90 family of protein chaperones. This family plays essential roles in the folding, maturation and activity of many proteins that are involved in signal transduction and transcriptional regulation. Among the number of proteins that are known to interact with Hsp90 are glucocorticoid receptors [5], Akt/Protein kinase B and Raf-1 [6], the tumor suppressor protein p53 [7] and NOS family members [8]. Despite being a family of proteins extensively studied in other fields such as cancer [9], little is known about the role of Hsp90 in chondrocyte biology. In the present work we identify TNF-α and nitric oxide donors as other positive modulators of Hsp90β, indicating a role of this chaperone in mediating key processes that take place in OA. Furthermore, we investigated the effect of Hsp90β inhibition on nitric oxide production by these cells, and found how knock-down of Hsp90β gene expression with small interfering RNA (siRNA) reduces NO-induced chondrocyte death.

## Methods

### Reagents

Culture media and fetal calf serum (FCS) were from Gibco BRL (Paisley, UK). Culture flasks were purchased from Costar (Cambridge, MA, USA). Unless indicated, the rest of chemicals and enzymes were obtained from Sigma-Aldrich (St. Louis, MO). Antibodies against human Hsp90β (sc-1057), α-tubulin (sc-5286), the peroxidase-conjugated secondary antibodies and the FITC-conjugated anti-goat secondary antibody were from Santa Cruz Biotechnology (Sta. Cruz, CA, USA).

### Cartilage procurement and processing

Macroscopically normal human knee cartilage from adult donors from both genders (mean age 60.3 years; age range 54-65 years) without history of joint disease was provided by the Tissue Bank and the Autopsy Service at Hospital Universitario A Coruña. Osteoarthritic cartilage was obtained from patients diagnosed with OA according to the American College of Rheumatology (ACR) criteria, which underwent joint surgery (mean age 64.6 years; age range 52-71 years). Knee radiographs from the OA participants were classified as grade IV according to the Kellgren and Lawrence (K/L) scoring system. All patients have signed the informed consent and the project was approved by the Regional Ethical Committee from Galicia (Spain). Once cartilage surfaces were rinsed with saline, scalpels were used to cut parallel sections 5 mm apart, vertically from the cartilage surface onto the subchondral bone. These cartilage strips were then cut-off from this bone, and the tissue was incubated with trypsin at 37°C for 10 minutes. After removing trypsin solution, the cartilage slices were treated for 12-16 h with type IV clostridial collagenase in Dulbecco's modified Eagle's medium (DMEM) with 5% FCS in order to release cartilage cells.

### Primary culture of chondrocytes

Chondrocytes were recovered and plated at high density (4 × 10^6 ^per 162-cm^2 ^flask) in DMEM supplemented with 100 units/mL penicillin, 100 μg/mL streptomycin, 1% glutamine and 10% FCS. The cells were incubated at 37°C in a humidified gas mixture containing 5% CO_2 _balanced with air. Chondrocytes were used at weeks 2-3 at confluency in primary culture. Cell viability was assessed by trypan blue dye exclusion.

### Protein sample preparation

Chondrocytes (3-5 × 10^6 ^cells) were recovered from culture flasks by trypsinization and washed twice in a saline buffer containing 130 mM NaCl, 5 mM KCl, 2.5 mM Tris HCl (pH 7.5) and 0.7 mM Na_2_HPO_4_. Cells were then transferred to microfuge tubes, where cell pellets were solubilized by vortexing and one hour incubation with gentle agitation in 200 μl of an isolectric focusing-compatible lysis buffer containing 8.4 M urea, 2.4 M thiourea, 5% cholamidopropyl diethylamoniopropane sulfonate (CHAPS), 1% carrier ampholytes (IPG Buffer pH 3-10 NL), 0.4% Triton X-100 and 2 mM dithiothreitol (DTT).

For protein quantification, 10 μl of the protein extract were diluted 10x with water and precipitated for at least 1 h with 0.02% sodium deoxycholate and 10% trichloroacetic acid. The precipitate was washed once with 2 volumes of ice-cold acetone, allowed to dry, and solubilized in alkaline SDS (5% SDS, 0.1 N NaOH). 5 to 10 μl of this sample were employed to quantify total chondrocytic proteins in each lysate by the BCA technique (Pierce Perbio, Rockford, IL, USA).

### Cell viability assay

Cell viability was evaluated by Trypan blue dye exclusion throughout the work. Nevertheless, due to the high amount of NO accumulation observed in chondrocytes after Novobiocin treatment, we decided to quantify cell viability with a more accurate method in these experiments. The MTS [3-(4,5-dimethylthiazol-2-yl)-5-(3-carboxymethoxyphenyl)-2-(4-sulfophenyl)-2Htetrazolium, inner salt] assay was employed with this aim, using the CellTiter 96^® ^AQueous Non-Radioactive Cell Proliferation Assay (Promega, Wisconsin, USA) kit and used following the manufacturer's instructions. Chondrocytes were seeded into 96-well plates at a density of 2 × 10^3 ^per well (100 μl). This cell number was empirically determined for our cells by performing a cell titration assay. Briefly, human chondrocytes (0.5 × 10^3 ^- 1 × 10^4^) in DMEM supplemented with 10% FBS were seeded onto a 96-well plate. The medium was allowed to equilibrate for 24 hours, and then 10 μl of combined MTS/PMS (phenazine methosulfate) solution were added to each well. After 3 h at 37°C in a humidified, 5% CO2 atmosphere, the absorbance at 490nm was recorded using an ELISA plate reader (Labsystems Multiskan Plus Plate Reader). Once the optimal cell number was determined (near the low end of the linear range of the assay), chondrocytes were treated with the following stimuli: vehicle control (DMEM), IL-1β 5 ng/mL, and Novobiocin at 100, 500 and 1000 μmol/L in presence or absence of IL-1β. Wells with serum-free medium were used as negative control. The cells were treated for 48 h. 3 h before each of the desired time points, 10 μl of the MTS reagent was added into each well and cells were further incubated at 37°C. The absorbance was detected at 490nm. All the experiments were repeated four times.

### Western blotting

Western blotting was performed according to standard procedures. 20 μg of protein were loaded and resolved on standard 10% polyacrylamide SDS-PAGE gels. Separated proteins were then electroblotted onto polyvinylidene difluoride membranes (Millipore Co, Bedford, MA, USA). Equivalent loadings were verified by Ponceau Red staining after transference. Membranes were blocked in Tris-buffered saline, pH 7.4, containing 0.1% tween-20 (TBST), and 5% non-fat dried milk for 60 minutes at room temperature. The blots were then hybridized overnight at 4°C with antibodies against Hsp90β (1:1000), and α-tubulin (1:5000). All antibodies were diluted in TBST with 2% non-fat milk. After extensive washing with TBST, immunoreactive bands were detected by chemiluminiscence using the correspondent peroxidase-conjugated secondary antibodies and ECL detection reagents (GE Healthcare), and digitized using a LAS 3000 image analyzer. Quantitative changes of protein were evaluated with ImageQuant 5.2 software (GE Healthcare).

### Induction and measurement of apoptosis

Apoptosis was induced by incubation of chondrocytes with sodium nitroprusside (SNP), or N-ethyl-2-(1-ethyl-2-hydroxy-2-nitrosohydrazine) ethanamine (NOC-12) at 0.5-1 mM in serum free DMEM for 24 hours. Cellular DNA content was assessed as described previously by flow cytometry [10]. For this purpose, 2 × 10^5 ^cells were cultured in 6-well plates and treated as appropriate. Then, cells were spun and resuspended in a solution containing 1 mg/ml propidium iodide (PI) (Sigma) in PBS. Then, they were incubated at 4°C for 30 minutes in the dark and analyzed by flow cytometry on a FACSCalibur (BD Biosciences, San Jose, CA, USA) using a 560 nm dichromatic mirror and a 600 nm band pass filter. The percentage of cells with decreased DNA staining (composed of apoptotic cells resulting from either fragmentation or decreased chromatin) of a minimum of 10,000 cells per experimental condition was counted. The data are expressed as the percentage of hypodiploid (apoptotic) nuclei. Cells with a very low DNA content, in which the type of cell death could not be ascertained, were excluded from the analysis.

The apoptotic response was also measured in the silencing experiments by Cell Death Detection ELISA^PLUS ^(enzyme-linked immunosorbent assay) (Roche Diagnostics, Mannheim, Germany, cat. N° 11774425001), following manufacturer's instructions and employing 2 × 10^4 ^cells seeded in 48-well plates. This kit is used for the quantitative *in vitro *determination of cytoplasmic histone-associated DNA fragments (mono- and oligonucleosomes) after induced cell death.

### Transfection of small interfering RNA (siRNA)

For the silencing experiments we used a pre-plated transfection procedure. Approximately 72 hr before transfection, 8 × 10^4 ^healthy adherent cells were trypsinized and plated in 12-well plates, growing them in normal medium (DMEM, 10% FCS, 1% P/S, 1% gentamicin) until they reach 80% confluency after 48 hr. Then, medium was replaced with antibiotics-free DMEM. Cells were transfected 24 hours later, following manufacturer's instructions with minor modifications. Briefly, we used 7 μL/mL of siPORT *Amine *Transfection Agent (Ambion, cat. N° AM4502) and the final RNA concentration was 30 nM for HSPCB *Silencer^® ^*Validated siRNA (Ambion, ref. AM51331). Controls were cells transfected without siRNA. Preliminary experiments determined that an incubation time of 72 h post-transfection was required to obtain an optimal level of HSPCB silencing. The transfected cells were treated with SNP and NOC-12, which served as apoptosis positive controls, and cell viability was assessed by trypan blue dye exclusion.

### RNA isolation and real-time PCR assays

Total RNA was isolated from chondrocytes using Invisorb Mini Kit (Invitek, Berlin, Germany), following manufacturer's instructions. Whole RNA was treated with DNase (Invitrogen), and its concentration was determined by spectrophotometry. 1 μg of RNA from each sample was reverse-transcribed in a final volume of 20 μL using the Transcriptor First Strand cDNA Synthesis Kit (Roche Applied Science). cDNA synthesis was performed at 55°C for 30 minutes followed by a final step of 5 minutes at 85°C for inactivating the reverse transcriptase. Tubes were finally stored at -20°C until PCR analyses.

Primers for HSP90B and HPRT1 (housekeeping gene) were intron-spanning designed using the Universal Probe Library tool available at Roche website (http://www.roche-applied-science.com). Primer sequences were as follows: HSP90B forward, 5'-cgttgctcactattacgtataatcct-3'; HSP90B reverse, 5'-tgcctgaaaggcaaaagtct-3' (108 bp product); HPRT1 forward, 5'-tgaccttgatttattttgcatacc-3'; HPRT1 reverse, 5'-cgagcaagacgttcagtcct-3' (102 pb product).

Real-time PCR was performed in a LightCycler 480 instrument (Roche Applied Science), with 20 μL reactions containing 10 μL LightCycler 480 SYBR Green I Master, 7 μL bidistilled water, 0.5 μL (0.5 μM) each primer and 2 μL cDNA as PCR template. Cycling parameters were 95°C for 10 minutes to activate DNA polymerase, followed by 45 cycles of 95°C for 10 seconds, 60°C for 10 seconds and a final extension of 72°C for 10 seconds. Detection of fluorescence was carried out at the end of each extension step. After amplification, a melting curve was acquired by heating to 95°C for 5 seconds, cooling to 70°C for 1 minute and slowly heating to 95°C with a continuous fluorescence data collection of 10 acquisitions per°C.

PCR data were analyzed using REST (Relative Expression Software Tool) software, which provides statistical information suitable for comparing groups of treated versus untreated samples while taking into account issues of reaction efficiency and reference gene normalisation.

### Indirect immunofluorescence

Chondrocytes were seeded at 5 × 10^4 ^cells per chamber in an 8-chamber slide. Cytokine stimulation was carried out on the chambers with IL1-β (5 ng/ml) or TNF-α (10 ng/ml) for 48 h. Then, media was removed and cells were fixed with acetone for 10 minutes at 4°C. After washing with PBS, anti-Hsp90β primary antibodies were applied at 1:50 dilution in PBS, and cells were incubated overnight in a humidified box at 4°C. After further washing, a FITC-conjugated anti-goat secondary antibody was used at 1:20 dilution for 1 h. Fluorescence microscopy was carried out in a Leica DMLS microscope. Quantification of the emitted fluorescence was performed with AnalySIS 5.0 software (Olympus Biosystems, Hamburg, Germany). Nuclei staining was performed with 4,6-dianidino-2-phenylindole dihydrochloride (DAPI, 2 mg/mL) for 30 minutes at 37°C.

### Determination of nitric oxide levels by nitrite quantification

For the evaluation of nitric oxide production by chondrocytes, 5 × 10^4 ^cells were placed onto culture 96-well plates and allowed to adhere for 24 h. All conditions were set by duplicate. Nitric oxide production was stimulated by the addition of 5 ng/ml IL-1β, and the Hsp90 inhibitors Geldanamycin (GA) and Novobiocin (NB) (Alexis Biochemicals, Lausen, Switzerland) were added at 1, 10, 25 and 50 nM (GA), or 100, 500 and 1000 μM (NB). Then, supernatants were collected and total nitrite released in cell culture medium was measured by the Griess method [11], using sodium nitrite as standard. Data were expressed as μM nitrites (NO_2_^-^) per number of cells per time.

### Statistical analysis

The data are expressed as the mean (SEM) from n determinations or as representative results, as indicated. The statistical software program, SPSS, was used to perform the analysis of variance. Differences were considered to be significant at *p *< 0.05.

## Results

### IL-1β and TNF-α increase the expression of Hsp90β in OA chondrocytes

**Figure 1:**
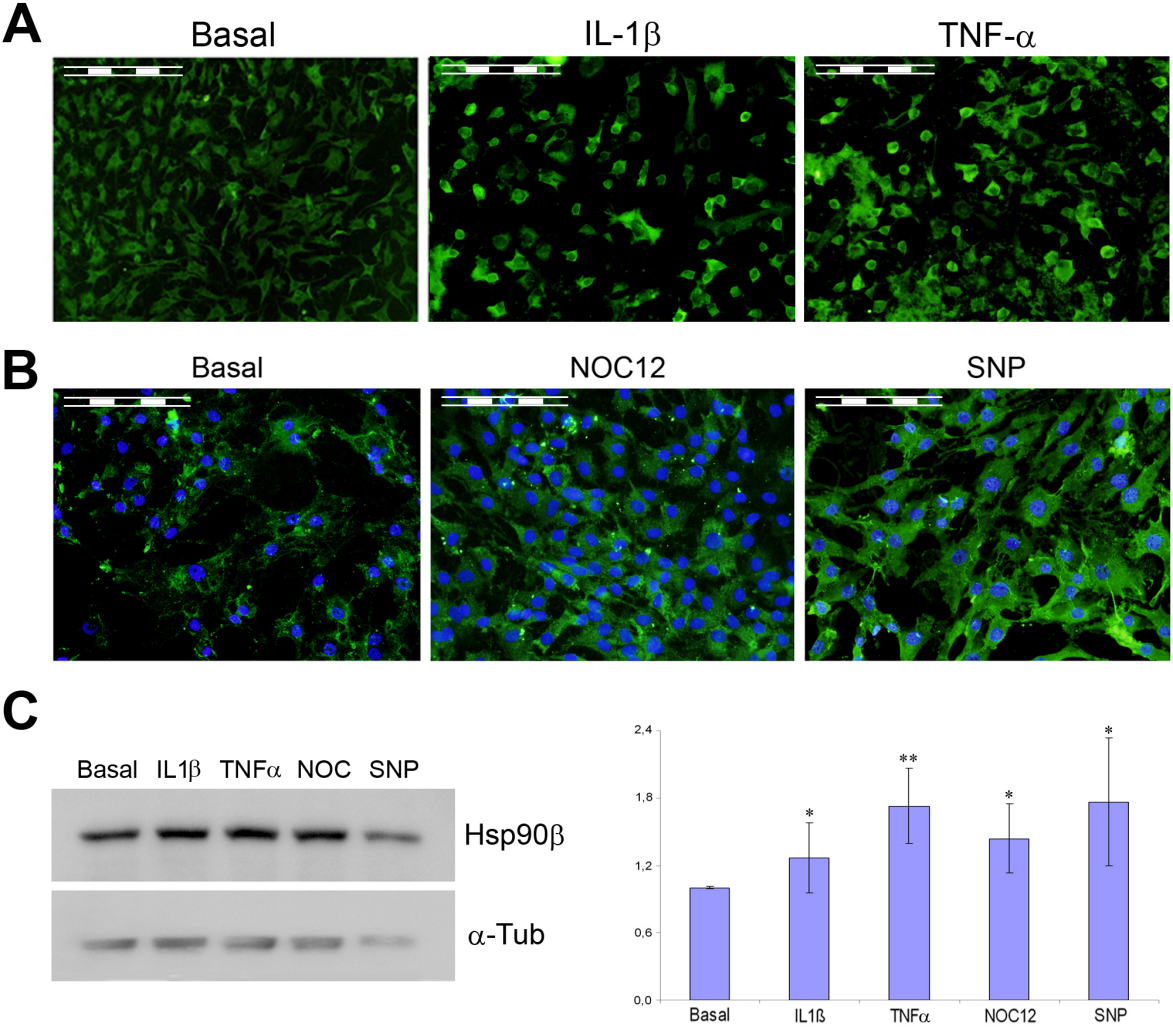
**Hsp90β is modulated by proinflammatory cytokines and NO donors in chondrocytes**. A) and B) Indirect immunofluorescence (IFI) analyses showing: A) the IL-1β- and TNFα- dependent increase of Hsp90β protein level; B) the increase of Hsp90β (green) after cellular stimulation with NOC-12 and SNP. Cellular nuclei, stained with DAPI, display a blue colour. Scale bars correspond to 100 μm. C) Western blot analysis on whole chondrocyte extracts stimulated for 48 h. α-Tubulin was used as loading control for normalization purposes. A representative image of the blots is shown in the left, together with the semiquantitative data obtained by densitometric analysis of the blots and normalized with the tubulin signal (on the right). Data are expressed as arbitrary volume units, and represent the mean (±SE) of four independent experiments (*, p < 0.05 and **, p < 0.01 vs basal).

In a previous study [4] performed by a gel-based proteomic analysis, we detected the beta isoform of the chaperone Hsp90 (Hsp90β) as significantly increased in OA cells (4.52-fold). OA cartilage is characterized by increases of the catabolic program which are induced by proinflammatory stimuli. Therefore, to further investigate the putative positive modulators of Hsp90β in OA, we now tested if the presence of proinflammatory cytokines such as Interleukin-1β (IL-1β) or Tumor Necrosis Factor-α (TNF-α) could have any effect on the amount of this protein in cultured normal chondrocytes. We stimulated the cells for 24 or 48 h with 5 ng/ml IL-1β or 10 ng/ml TNF-α. As shown in the indirect immunofluorescence images that are illustrated in Figure 1A, both cytokines were capable of increasing Hsp90β abundance in the cytoplasm of cultured chondrocytes. These results suggest a role of Hsp90β in mediating the cellular response against those inflammatory processes, driven by IL-1β, that participate in OA pathogenesis.

One of the reported consequences of the stimulation of articular chondrocytes by inflammatory cytokines is the increase in nitric oxide synthesis from these cells [12]. To further investigate if nitric oxide has any direct effect on Hsp90β protein abundance, we also carried out indirect immunofluorescence analyses after stimulating the cells with different NO donors. With this aim, we treated the chondrocytes with 0.5 to 2 mM NOC-12 or SNP for 24 hours. As shown in Figure 1B, both NO donors increase Hsp90β abundance in chondrocytes, being this increase higher with SNP.

The immunofluorescence results were confirmed with a more quantitative assay. We performed Western blot tests on whole chondrocyte extracts obtained from cells that were treated for 48 h with the different stimuli. The blots were densitometrically analyzed, being their intensities normalized against a housekeeping protein, α-tubulin. As shown in Figure 1C, all of the studied compounds increase Hsp90β protein expression in chondrocytes, from 1.31-fold (IL-1β) to 1.76-fold (SNP).

### Hsp90β inhibition reduces NO synthesis in chondrocytes

**Figure 2:**
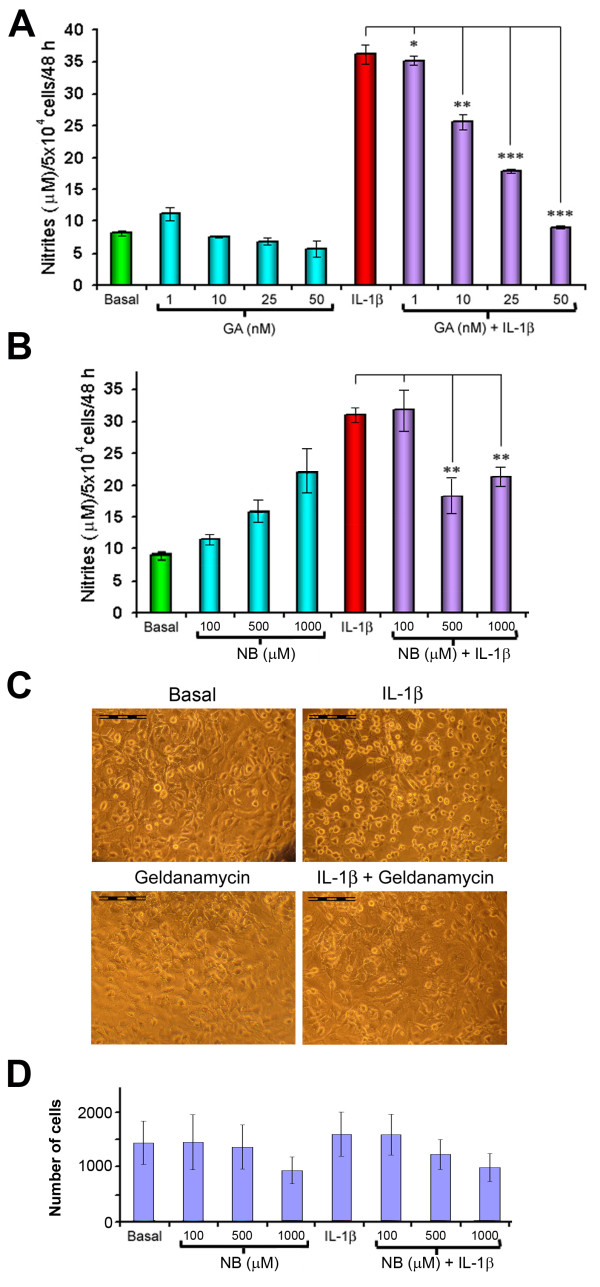
**Hsp90β is involved in the synthesis of NO caused by IL-1β stimulation of chondrocytes**. Effect of Hsp90 inhibitors Geldanamycin (GA) (A) and Novobiocin (NB) (B) on the production of nitrites by IL-1β-treated chondrocytes. Data are expressed as mM of nitrites present in the supernatant, and represent the mean (±SE) of three independent experiments in triplicate (*, p < 0.05; **, p < 0.01 and ***, p < 0.005 vs basal). C) Phase contrast microscopy images illustrate how the Hsp90 inhibitor Geldanamicyn (GA) does not affect cellular viability and counteracts the morphological modifications caused by IL-1β in cultured chondrocytes. Scale bars correspond to 100 μm. D) Cell viability of chondrocytes treated with IL-1β and Novobiocin, measured by the MTS assay.

To investigate if Hsp90β might participate in NO signalling in chondrocytes and the possible role of this chaperone in their response after cytokine stimulation, we measured nitrite production in the supernatants of chondrocytes treated with the cytokine IL-1β and different amounts of the well-known Hsp90β inhibitors Novobiocin (NB) and Geldanamycin (GA) [13]. We observed with both inhibitors a dose-dependent decrease of those high nitrite levels induced by the cytokine, from 37.28 ± 1.9 down to 8.85 ± 0.23 μM NO_2_^- ^per 5 × 10^4 ^cells per 48 h of production, being the differences statistically significant (all of them with p < 0.05) with GA (Figures 2A and 2B).

We also exposed the cells to IL-1β and the Hsp90β inhibitor GA for their phase contrast microscope observation. The cellular morphology of these cells (exemplified in Figure 2C) reveals those striking morphological changes caused by the cytokine in cultured chondrocytes, and how the addition of the Hsp90β inhibitor GA has no effect on cellular viability (as determined by Trypan blue dye exclusion) and even restores the normal chondrocyte morphology in IL1β-treated cells.

Finally, a significant NO accumulation was observed with NB treatment alone, which rises dose-dependently from 11.2 μM NO (with 100 μM NB) to 22.1 μM (with 1 mM NB). Therefore, we performed a quantitative assay of the chondrocyte viability under these conditions using the MTS reagent. As shown in Figure 2D, no significant reduction in cell viability is observed after treatment with up to 100 μM NB, whereas the presence of the inhibitor at 1 mM concentration decreases chondrocyte viability a 35% in average.

### Hsp90 inhibitors prevent cellular death caused by NO donors in chondrocytes

**Figure 3:**
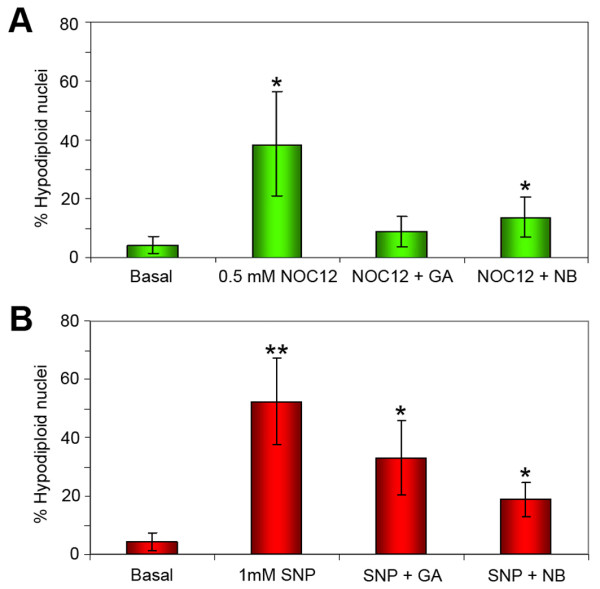
**Hsp90β inhibitors prevent chondrocyte apoptosis induced by nitric oxide donors**. Flow cytometry analysis of chondrocytes treated with NOC-12 (A), SNP (B) and the Hsp90β inhibitors GA and NB. Apoptosis is expressed as percentage of hypodiploid nuclei present in the population, and represent the mean (±SE) of four independent experiments by triplicate. Error bars indicate standard deviation of the mean (*, p < 0.05 vs basal).

It is well known that NO regulates catabolic processes in chondrocytes, and can induce apoptosis [14]. Taking into account the effect of Hsp90 inhibitors on NO production from chondrocytes, we tested if these compounds may exert any effect on the cellular death induced by NO donors. We stimulated the cells with NOC-12 (0.5 mM, Figure 3A) and SNP (1 mM, Figure 3B) alone or in combination with the Hsp90 inhibitors GA or NB, and the apoptosis levels were measured by flow cytometry. As shown in Figure 3, treating the chondrocytes with NOC-12 or SNP provokes the presence of a 38.31 ± 19.6% and 52.07 ± 15.4% of apoptotic cells in the population, respectively. Addition of Hsp90 inhibitors reduces the cellular death down to a 9.02 ± 5.3% (NOC-12 with GA).

### Specific Hsp90β silencing diminishes the cellular death induced by NO donors

**Figure 4:**
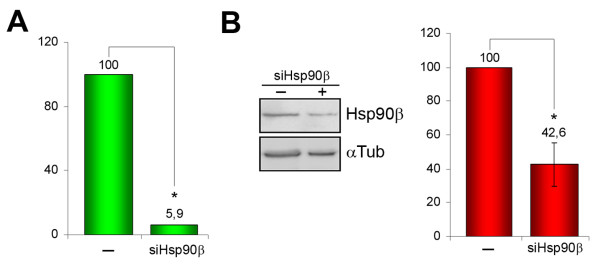
**Effect of gene silencing on Hsp90β transcription and protein abundance**. Chondrocytes were transfected with the specific Hsp90β small interfering RNA. Total RNA and cellular proteins were extracted from control and transfected cells. A) Real-time PCR assays confirm the decrease of HSP90B gene expression in the presence of the siRNA. Data are expressed relative to gene expression without siRNA (*, p < 0.0001; n = 3). B) Representative western blot that illustrates the effect of siHsp90β at the protein level. Semi-quantitative data, obtained by densitometric analysis of the blots and normalization of each lane using α-tubulin signal, are shown at the right (*, p < 0.01; n = 3).

**Figure 5:**
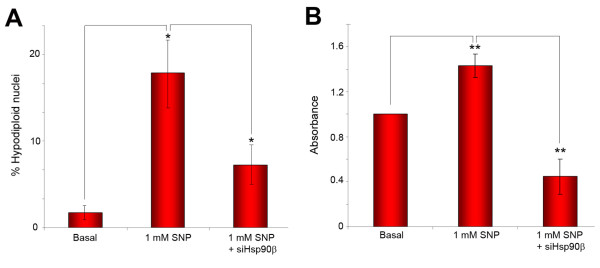
**Hsp90β silencing diminishes the cellular death of chondrocytes induced by nitric oxide**. Determination of apoptosis levels by flow cytometry (A) or ELISA (B) in chondrocytes exposed to 1 mM SNP. In the flow cytometry assays, data are represented as percentage of apoptotic (hypodiploid) nuclei, whereas the ELISA experiments show absorbance units relative to basal conditions (*, p < 0.05; **, p < 0.01 vs basal or SNP-treated, as indicated). Data represent the mean (±SE) of three independent experiments performed in duplicate.

Hsp90 inhibitors have shown to be related with a number of cellular processes, being in some cases rather unspecific. In order to confirm if the results previously demonstrated are specific of Hsp90β inhibition, we determined whether Hsp90β silencing affected the cellular death levels of chondrocytes exposed to NO donors. With this aim, chondrocytes were transfected with Hsp90β siRNA. Output of the silencing on Hsp90β transcriptional (A) and protein (B) levels are shown in Figure 4. Gene silencing with siRNA provoked a 17-fold decrease of Hsp90β gene expression, as demonstrated by real-time PCR (Figure 4A), and more than a 2-fold reduction of protein abundance in chondrocytes (Figure 4B). Transfected cells (n = 3) were then cultured and treated for 24 h in the presence or absence of 1 mM SNP. Apoptosis levels were determined by flow cytometry and ELISA. Data obtained revealed that Hsp90β silencing reduces the cellular death provoked by SNP in chondrocytes 2.4-fold, when determined by flow cytometry (from 17.41 ± 8.9% to 7.36 ± 4.7% hypodiploid nuclei, n = 3, p < 0.05, Figure 5A), and 3.2-fold when determined by ELISA (from 1.43 ± 0.14 to 0.45 ± 0.20 absorbance units, n = 3, p < 0.01, Figure 5B). Similar results were obtained with NOC-12, although they were not statistically significant with the number of experiments analyzed (data not shown).

## Discussion

We previously showed the increased abundance of the chaperone Hsp90β in OA chondrocytes grown in monolayer culture, when compared to normal cells [4]. In this work, our aim was to gain insight into the modulation of Hsp90β in human articular chondrocytes and the possible outputs of an increase of this chaperone in this type of cells.

The cytosolic Hsp90β is a calcium-binding protein that belongs to the family of 90 kDa protein chaperones [15]. It is involved in the folding, activation and assembly of several proteins. Our finding of an increase of this protein in osteoarthritic chondrocytes, along with other chaperones such as Grp78 or Grp94 [4], points to an essential role of the stress response in OA pathogenesis that should be studied in more detail. Therefore, we have now demonstrated that the presence of Hsp90β in chondrocytes is increased after stimulating the cells with proinflammatory cytokines involved in cartilage destruction, such as IL-1β or TNF-α, and also by NO-induced stress.

In contrast to our data, Hsp90β has been recently found to be a novel regulatory factor of MMP-13 expression in osteoarthritic chondrocytes [16]. In this work, authors describe how silencing Hsp90β significantly increased MMP-13, which indicates a negative modulation driven by the chaperone. Moreover, they show how the addition of IL-1β decreased Hsp90β production. These results are opposed to our data and those by Boehm et al., who previously reported that Hsp90 inhibition (of both α and β forms) blocked IL-1β-induced up-regulation of MMP-13 in equine articular chondrocytes [17]. Moreover, most recently Kimura et al. identified a promising compound for OA therapy that achieved the above mentioned MMP-13 production blockage [18], and subsequently determined that this compound acts as a client-selective Hsp90 inhibitor [19]. This divergence might be explained by the different quantities of IL-1β employed to induce metalloprotease production, as Fan et al. use 100-fold less amount of cytokine than the others.

Moreover, the use of chemical inhibitors can be controversial. Traditional Hsp90 inhibitors have been extensively studied for oncology therapy [20], and small molecule inhibitors of Hsp90 have been described to affect inflammatory disease pathways in models of rheumatoid arthritis [21]. Both Geldanamycin (GA), which binds to the N-terminal ATP-binding site of the protein [22], and Novobiocin (NB), which binds to a second ATP-binding site on the C-terminal domain [23] are global Hsp90 inhibitors: that is, they are known to block both Hsp90 isoforms (α and β), which share about 80% homology, and maybe other factors. In this sense, GA has proven to inhibit the ATPase activity of TRAP1, a mitochondrial homolog of Hsp90 [24] that was recently identified as increased in OA mitochondria [25]. In the present work, we have employed these compounds to screen the effects of low amounts of this chaperone in chondrocytes subjected to cytokine and NO stress in an attempt to mimic an OA status. Then, in order to search for the specific effect of Hsp90β, we have silenced this β isoform with siRNA and can therefore conclude that this protein really participates in the NO-induced apoptotic process of chondrocytes.

Evidence suggests that Hsp90 is an allosteric enhancer of inducible nitric oxide synthase (iNOS) activity, and that binding of Hsp90 to this enzyme is required for iNOS activity [26]. IL-1β stimulates cartilage ECM degradation in OA, in part through up-regulation of iNOS, which increases NO production [27,28]. NO has been shown to regulate catabolic reactions in chondrocytes, being able to induce apoptosis [14]. A number of NO donors have been described to reduce respiration and ATP generation, which suggests a contribution to cartilage ECM loss and mineralization [29]. With this background, the decreased synthesis of NO that we observed after Hsp90 inhibition is an interesting positive outcome that points to Hsp90 as putative target for OA.

Moreover, Hsp90 plays a well-known role as major repressor of heat shock transcription factor HSF1 [30], whose activation increases the synthesis of several cytoprotective proteins [31]. Inhibition of Hsp90 (and also stress or heat shock conditions) releases HSF1 from the Hsp90 complex, which results in its activation and translocation to the nucleus, where it initiates a protective response with a transcriptional program linked to cellular adaptation and survival [32], and manifested in the production of proteins with anti-apoptotic or protein folding functions. As a proof of act, it has been demonstrated very recently that HSF1 inhibits H_2_O_2_-induced apoptosis via down-regulation of reactive oxygen species [33]. With the same reasoning, it is not tempting to speculate that HSF1 activation might also drive the protective mechanisms working against NO-induced apoptosis when Hsp90β is inhibited. All these data shed light again on the usefulness of Hsp90 as therapeutic target for OA.

Given the broad spectrum of cellular roles played by this chaperone, the question arises as to whether Hsp90 global inhibition would be efficient in the treatment of OA. Recent investigations such as those reported above [19], which employ client-selective Hsp90 inhibitors, might have the highest therapeutic potential, in an attempt to reduce their pleiotropic abilities and drive them to a specific area of Hsp90 effects, such as those related with NO synthesis and protecting chondrocyte from apoptosis.

## Conclusions

The present work depicts the effect of Hsp90 inhibition in the modulation of NO production by human OA chondrocytes, and also in protecting these cells from NO-provoked death. Additional evaluation is needed to support the therapeutic use of selective Hsp90 inhibitors in OA, and further studies on the role of Hsp90 in diverse OA-related pathogenesis processes would help to attain this objective.

## List of abbreviations used

ATPase: adenosine triphosphatase; BCA: bicinchoninic acid assay; cDNA: complementary DNA; CHAPS: cholamidopropyl diethylamonio propane sulfonate; DAPI: 4',6-diamidino-2-phenylindole; DMEM: Dulbecco's modified Eagle's medium; DNA: deoxyribonucleic acid; DTT: dithiothreitol; ECL: enhanced chemiluminescence; ECM: extracellular matrix; ELISA: enzyme-linked immunosorbent assay; FCS: fetal calf serum; FITC: fluorescein isothiocyanate; GA: geldanamycin; Grp78: glucose regulated protein 78; Grp94: glucose regulated protein 94; Hsp90: heat shock protein 90; Hsp90β: heat shock protein 90 beta; HSPCB: heat shock protein 90 beta; IL-1β: inteleukin-1 beta; iNOS: inducible nitric oxide synthase; IPG: immobilized pH gradient; MMP-13: matrix metalloprotease 13; NB: novobiocin; NO: nitric oxide; NO2-: anion nitrite; NOC-12: N-ethyl-2-(1-ethyl-2-hydroxy-2-nitrosohydrazino) ethanamine; NOS: nitric oxide synthase; OA: osteoarthritis; P/S: penicillin/streptomycin; PAGE: polyacrylamide gel electrophoresis; PBS: phosphate buffered saline; PCR: polymerase chain reaction; PI: propidium iodide; RNA: ribonucleic acid; SDS: sodium dodecyl sulphate; SEM: standard error of the mean; siRNA: small interfering RNA; SNP: sodium nitroprusside; TBST: tris-buffered saline tween-20; TNF-α: tumor necrosis factor alpha; TRAP1: TNF receptor-associated protein 1

## Competing interests

The authors declare that they have no competing interests.

## Authors' contributions

VC carried out the experimental work and analysed the data. MCDA helped in collecting and processing protein samples, participated in GA and NB experiments and helped in statistical data analysis. NO provided cartilage samples for the study. CRR participated in study design, interpretation of data and drafted the manuscript. FJB conceived and coordinated the project and revised the manuscript. All authors read and approved the final manuscript.

## Pre-publication history

The pre-publication history for this paper can be accessed here:

http://www.biomedcentral.com/1471-2474/12/237/prepub
